# Pain and Inflammation Management in Older Adults: A Brazilian Consensus of Potentially Inappropriate Medication and Their Alternative Therapies

**DOI:** 10.3389/fphar.2019.01408

**Published:** 2019-12-02

**Authors:** Fabiane Raquel Motter, Sarah Nicole Hilmer, Vera Maria Vieira Paniz

**Affiliations:** ^1^Postgraduate Program in Collective Health, University of Vale do Rio dos Sinos (UNISINOS), São Leopoldo, Brazil; ^2^Kolling Institute of Medical Research, Royal North Shore 83 Hospital, St. Leonards, NSW, Australia

**Keywords:** inappropriate prescribing, potentially inappropriate medications list, pain management, deprescriptions, aged, Brazil

## Abstract

**Purpose:** The aim of the present study was to develop and validate a Potentially Inappropriate Medications (PIM) list and alternative therapies for treatment of pain and inflammation in older people adapted to the Brazilian context.

**Methods:** A preliminary PIM list suitable for the Brazilian market was developed on the basis of three published international PIM lists [Beers 2015, Screening Tool of Older People’s Potentially Inappropriate Prescriptions - 2015, European Union (7) PIM list]. We used the modified Delphi technique (two-round) to validate concerns of use and alternative therapies related to PIM for treatment of pain and inflammation in older adults ≥65 years in Brazil. The panel involved nine Brazilian experts in geriatric pharmacotherapy. All items with mean Likert scale score ≥4.0 (agree) and the lower limit of 95% confidence interval ≥4.0 were considered validated in this study.

**Results:** At the end of the consensus process, 94 (65.3%) items of 144 were validated. In total, consensus was reached for 33/35 (94.3%) concerns about drugs that should be avoided in older patients regardless of diagnosis, for 22/23 (95.7%) concerns about drugs that should be avoided in older patients with specific conditions or diseases, for 11/23 (47.8%) with special considerations of use, and for 28/63 (44.4%) of therapeutic alternatives.

**Conclusion:** Although these criteria are not designed to replace clinical judgement, PIM and alternative therapies lists can be useful to inform prescribers, pharmacists, and health care planners and may serve as a starting point for safe and effective use of medications in older people.

## Introduction

The process of aging in a population is accompanied by a rising prevalence of chronic and degenerative diseases and, consequently, a higher incidence of conditions characterised by pain and inflammation (Horgas, 2017). Studies show that the prevalence of chronic pain among elderly people in the community ranges from 21.5% to 65.0%, depending on the study population, the sampling method, the interview method, and the definition of “chronic pain” (Hairi et al., 2013; Eggermont et al., 2014; Jackson et al., 2015; Leao Ferreira et al., 2016; Larsson et al., 2017; Cimas et al., 2018; Liberman et al., 2018; Dahlhamer et al., 2018).

Pain and inflammation management in older people is a challenge for health professionals. Older persons often have age-related physiological changes and a high number of comorbidities, and undergo a number of therapies, which increase the risk of adverse drug effects, making it difficult to establish a balance between the benefits and risks of medications used in this population (Hilmer et al., 2007; McLachlan et al., 2011). In addition, some of the most commonly prescribed medications for the treatment of pain and inflammation can confer significant risks on older adults and have been associated with the occurrence of adverse events such as falls, fractures, gastrointestinal bleeding, worsening of heart failure, cognitive impairment, and renal failure (Marcum and Hanlon, 2010; O’Neil et al., 2012).

Potentially inappropriate medication (PIM) is a term used to describe a medicine for which the risk associated with its use outweighs the potential benefits, especially when there are more effective alternatives available (Gallagher et al., 2008; Renom-Guiteras et al., 2015). Several explicit criteria have identified medications that are considered inappropriate for the treatment of pain and inflammation in older people (McLeod et al., 1997; Gallagher et al., 2008; Winit-Watjana et al., 2008; Holt et al., 2010; Kim et al., 2010; American Geriatrics Society Beers Criteria Update Expert P, 2012; Chang et al., 2012; Mann et al., 2012; Clyne et al., 2013; Renom-Guiteras et al., 2015; American Geriatrics Society Beers Criteria Update Expert P., 2015; Kim et al., 2015; O’Mahony et al., 2015; Oliveira et al., 2016). These tools were developed by expert consensus and provide an accessible resource for health professionals in different settings.

Despite the availability of information, PIMs continue to be prescribed and used as first-line medications in older people. The frequency of PIM is high across a variety of healthcare settings, including Brazil. According to Brazilian studies, 42–59% of older people use at least one PIM (Baldoni et al., 2014; Martins et al., 2015; Lutz et al., 2017; Nascimento et al., 2017). Some of most commonly PIMs are used for treating pain and inflammation. These results illustrate that more work is needed to improve the use of appropriate medications in older adults.

The first Brazilian consensus on PIMs was published in 2016 (Oliveira et al., 2016). Limitations reported by the authors included that the criteria were based on previous versions of Beers (2012) (American Geriatrics Society Beers Criteria Update Expert P, 2012) and STOPP (2008) (Gallagher et al., 2008) and did not incorporate therapeutic alternatives. Evidence-based clinical practice guidelines including the list of PIMs should be continuously updated to incorporate emerging or changing evidence as well as newly approved drugs in order to remain current in line with current evidence (Shekelle et al., 2012). In 2015, updates of Beers (American Geriatrics Society Beers Criteria Update Expert P., 2015) and STOPP (O’Mahony et al., 2015) and a new European list [EU(7)] were published (Renom-Guiteras et al., 2015). This last PIM list was based on several international PIM lists and contains suggestions for dose adjustments, special considerations of use and therapeutic alternatives.

Therefore, the aim of the present study was to develop and validate a PIM list for the treatment of pain and inflammation and their respective alternative therapies based on the three international PIM lists recently updated [Beers, STOPP, and EU(7) PIM list], applicable to Brazilian elderly individuals (Renom-Guiteras et al., 2015; American Geriatrics Society Beers Criteria Update Expert P., 2015; O’Mahony et al., 2015).

## Methods

We used the modified Delphi technique to validate concerns and alternative therapies related to PIMs for the treatment of pain and inflammation in older adults aged ≥65 years in Brazil. This process combines evidence from the literature and expert opinion, and has been successful in the development of previous explicit criteria for older people (Kim et al., 2010; Chang et al., 2012; Renom-Guiteras et al., 2015; Kim et al., 2015; Oliveira et al., 2016; Aliberti et al., 2018). In this method, the search for consensus is systematic. The experts assess the information, also called propositions, presented by the researcher(s) in the form of a previously formulated questionnaire. The questionnaire is based on the literature review of a research problem and presents a synthesis of the main discussions on the subject during rounds (Dalkey, 1969; Campbell and Cantrill, 2001).

The development of this study comprised of five steps: preparation of a preliminary PIM list, the plan for a modified Delphi study (elaboration of a data collection instrument and selection of an expert panel), two rounds of survey, and the summary of consensus.

### Preliminary List of PIM and Alternative Therapies for the Treatment of Pain and Inflammation

A systematic literature review was performed in order to identify possible screening tools for detection of PIMs published between January 1991 and April 2017 (Motter et al., 2018). After the review of 36 different tools, the list of PIMs related to pain and inflammation management was based on a combination of the relevant medicines from the updated Beers criteria (American Geriatrics Society Beers Criteria Update Expert P., 2015), updated STOPP criteria (O’Mahony et al., 2015), and EU(7) PIM list (Renom-Guiteras et al., 2015). These PIM lists are the most comprehensive and updated previously published lists. The original version of the PIM lists (in English) was translated into Brazilian Portuguese by two Brazilian researchers. The availability of medications and alternative therapies listed in the Brazilian market was confirmed by a medication database from the Brazilian National Health Surveillance Agency (National Agency of Sanitary Surveillance A, 2017). When a medication class was listed in a published PIM list, we identified all medications belonging to the class that were available in Brazil. The concerns about each medication/medication class were formulated using the information provided in the original list. The list of special considerations of use and alternative therapies was based on the EU(7) PIM lists (Renom-Guiteras et al., 2015).

The preliminary PIM list was organized by medication/medication class. In total, the list of 12 PIMs contained 104 items which involved: 35 concerns about medications/medication classes that should be avoided in older people regardless of diagnosis, 20 concerns about medications/medication classes that should be avoided in specific diseases or conditions, 19 dose adjustments and special considerations of use, and 30 possible therapeutic alternatives. In the second round, items suggested by experts during the first round could be added to the preliminary PIM list.

### Selection of Expert Panel

The survey of experts was carried out through an initial search of the Lattes platform on the National Council for Scientific and Technological Development (CNPq) website (National Council of Technological and Scientific Development, 2016) on the 10th and 30th of August, 2016, using the following descriptors: “geriatric” and “medications.” The members were primarily selected based on their expertise in the areas of geriatric medicine and/or clinical pharmacology. Additionally, their regional location was considered in order to provide national representation and to gain national perspective on these topics. We identified 47 potential participants (geriatricians and pharmacists) who were then invited by email which contained information on the study objective and a link to access the informed consent form. They were assured that participation in the consensus process was voluntary and confidential.

### First and Second Rounds

As the experts were based in disparate geographical locations across Brazil, an online two-round Delphi questionnaire was administered to facilitate efficient data collection; a link to the questionnaire on the Google Docs^®^ website was provided. The first round took place between January and May 2017, and the second round between May and June 2017. We asked experts to assess each item of the preliminary list using a five-point Likert scale that ranged from one point (strongly disagree) to five points (strongly agree). The experts were also offered the opportunity to add items and to suggest alternative treatments. All the respondents in the first-round questionnaire were invited to participate in the second-round questionnaire. The second round included items for which no consensus had been reached in the first round (see *Data Analysis* section) and any new item suggested by experts in the first round. Quantitative feedback (percentage rating) from the first round of the Delphi process was incorporated into the survey questionnaire for the second round. The expert panel was instructed to consider the feedback provided while re-scoring the items contained in the second-round questionnaire. For both rounds, reminder emails were sent as necessary to encourage participation.

### Data Analysis

The collected data were organized in an Excel spreadsheet and subsequently imported into STATA 12.0 statistical software (StataCorp LP, College Station, TX, USA). A descriptive analysis was performed, and the absolute and relative frequencies, means and 95% confidence interval (CI) of the study items were evaluated. At the end of the consensus process, only items with mean Likert scale ≥4.0 (agree) and lower limit of CI ≥4.0 were considered validated in this study. This cut-off point is similar to that used by Oliveira et al. (Oliveira et al., 2016) and higher than that used in another study with similar scale (Chang et al., 2012).

## Results

### Participants

Of the 13 experts who agreed to participate in the study, 10 were geriatricians and 3 were pharmacists. Among them, nine completed the first round while seven completed the second round. All respondents of the second round participated in first round. We were unable to identify which experts had left the panel, because the first round was conducted anonymously. At the end of the consensus process, all participants were geriatricians with more than 10 years of experience in geriatric medicine.

### First and Second Rounds

In the first round, experts reached consensus on 51/104 (49.0%) items in the preliminary tool. Among these, 26/51 (51,0%) items were concerns about medication/medication classes that should be avoided in older people regardless of diagnosis, 17/51 (33,3%) were concerns about medication/medication classes that should be avoided in specific diseases or conditions, and seven were special considerations of use. Only one possible alternative therapy reached consensus in this step.

After the first round, the items were revised in concordance with the comments from the experts. Four items were modified, and 40 new items were added. The majority (32/40; 82.5%) of items suggested by the experts were possible alternative therapies. Items for which consensus was not achieved were resubmitted for the second round (N = 53).

In the second round, experts evaluated 97 items. Among these, 44/97 (45.4%) reached consensus. One modified item was validated and replaced the original item. At the end of the consensus process, 94 (65.3%) items of 144 were validated. In total, consensus was reached for 33/35 (94.3%) concerns about drugs that should be avoided in older patients regardless of diagnosis, for 22/23 (95.7%) of concerns about drugs that should be avoided in older patients in specific conditions or diseases, for 11/23 (47.8%) of special considerations of use, and for 28/63 (44.4%) of therapeutic alternatives.

**Table 1** presents the medication/medication classes considered inappropriate for older people independent of their diagnosis and their respective average Likert scales and CIs. The expert panel classified nonsteroidal anti-inflammatory drugs (NSAIDs), muscle relaxants, colchicine, and opioids as PIMs regardless of the diagnosis in the study. **Table 1** also provides information about dose adjustments and special considerations for medication use and alternative therapies validated by experts in this study.

**Table 1:** Potentially inappropriate drugs for the older patients independent of diagnosis validated by expert consensus.

Inappropriate medication	Concern	Average of Likert scales (CI95%)^a^ from panel members	Dose adjustment/special considerations of use^i^	Alternative drugs and/or therapies^i^	Concern described in other PIM lists		
					Beers	STOPP^j^	Eu^l^ (7) - PIM list
**NSAIDs** **^b^** Diclofenac Etodolac Aceclofenac Piroxicam Lornoxicam Tenoxicam Meloxicam Ibuprofen Flurbiprofen Loxoprofen Mefenamic acid Celecoxib Etoricoxib Nimesulide Acetylsalicylic –acid Phenylbutazone Indomethacin	Very high risk of gastrointestinal bleeding, ulceration, or perforation, which may be fatal.	4.89 (4.63; 5.15) 4.63 (4.00; 5.25) 4.75 (4.16; 5.34) 4.75 (4.16; 5.34) 4.63 (4.00; 5.25) 4.67 (4.28; 5.05) 4.63 (4.00; 5.25) 4.67 (4.28; 5.05) 4.63 (4.00; 5.25) 4.63 (4.00; 5.25) 4.75 (4.16; 5.34) 4.56 (4.00; 5.11) 4.57 (4.07; 5.07)^d^ 4.67 (4.28; 5.05) 4.56 (4.00; 5.11) 4.78 (4.27; 5.29) 4.67 (4.12; 5.21)	Use with caution in older patients with hepatic insufficiency^c,d^.	Paracetamol; Dipyrone 500–1000 mg q6hr or q8/hrc,d; Non-pharmacological treatment (e.g., physiotherapy, acupuncture, thermotherapy, electrostimulation, and therapeutic massage)c,d	x^e^		x
Ketorolac		4.63 (4.00; 5.25)	Contraindicated in cases of advanced renal failure.				
Naproxen		4.67 (4.28; 5.05)	Start with lower dose and use reduced maintenance dose in older adults. Avoid if Creatinine Clearance <30 ml/min.				
Ketoprofen		4.78 (4.44; 5.12)	Start with lower dose and use reduced maintenance dose in older adults.				
**Indomethacin**	Indomethacin is more likely than other NSAIDsb to have adverse central nervous system effects.	4.63 (4.00; 5.25).		Paracetamol; Dipyrone 500–1000 mg q6hr or q8hr^c,d^; Non-pharmacological treatment (e.g., physiotherapy, acupuncture, thermotherapy, electrostimulation, therapeutic massage)^c,d^	x		x
**Ketorolac**	Increased risk of acute kidney injury in older adults.	4.63 (4.00; 5.25)	Contraindicated in cases of advanced renal failure.	Paracetamol; Dipyrone 500–1000 mg q6hr or q8hrc,d Non-pharmacological treatment (e.g., physiotherapy, acupuncture, thermotherapy, electrostimulation, therapeutic massage)^c,d^	x		x
**Ibuprofen**	Ibuprofen (>3 × 400 mg/day): increased risk of cardiovascular complications at higher doses ( >1200 mg/day), especially in cases of previous cardiovascular disease.	5.00 (5.00; 5.00)^d^		Paracetamol; Dipyrone 500–1000 mg q6hr or q8hr^c,d^ h^c,d^; Non-pharmacological treatment (e.g., physiotherapy, acupuncture, thermotherapy, electrostimulation, therapeutic massage)^c,d^			x
**Acetylsalicylic acid**	Acetylsalicylic acid (>325 mg): increased risk of bleeding due to prolonged clotting time, elevation of INR values or inhibition of platelet aggregation.	5.00 (5.00; 5.00)^d^		Paracetamol; Dipyrone 500–1000 mg q6hr or q8hr^c,d^ h^c,d^; Non-pharmacological treatment (e.g., physiotherapy, acupuncture, thermotherapy, electrostimulation, therapeutic massage)^c,d^			x
**Muscle relaxants** Carisoprodol Orphenadrine Baclofen Thiocolchicoside Cyclobenzaprine	Most muscle relaxants are poorly tolerated by older adults owing to their anticholinergic adverse effects, sedation, and increased risk of fractures; their effectiveness at dosages tolerated by older adults is questionable.	4.88 (4.58; 5.17) 5.00 (5.00; 5.00)^d^ 4.83 (4.40; 5.26)^d^ 4.75 (4.36; 5.14) 4.67 (4.12; 5.21)^d^		Non-pharmacological treatment (e.g., physiotherapy, acupuncture, thermotherapy, electrostimulation, therapeutic massage)^c,d^	x x		x x x
					x		x
**Colchicine**	Higher risk of toxicity in older adults, particularly in cases of existing renal, gastrointestinal infections, or cardiac disease.	4.67 (4.28; 5.05)	Reduce dose by 50% in older adults (>70 years old).	Paracetamol^d^; Dipyrone 500–1000 mg q6hr or q8hr^c,d^; Non-pharmacological treatment (e.g., physiotherapy, acupuncture, thermotherapy, electrostimulation, therapeutic massage)^c,d^			x
**Opioids**	Use of regular (as distinct from PRN) opioids without concomitant laxative confers a risk of severe constipation.	5.00 (5.00; 5.00)^d^	Reduce dose in cases of renal failure.	Paracetamol^d^; Dipyrone 500–1000 mg q6hr or q8hr^c,d^ h^c,d^; Non-pharmacological treatment (e.g., physiotherapy, acupuncture, thermotherapy, electrostimulation, therapeutic massage)^c,d^		x	
**Meperidine/Pethidine**	Risk of falls, fractures, confusion, dependency and withdrawal syndrome. Not effective oral analgesic in dosages commonly used. May have higher risk of neurotoxicity (including delirium) than other opioids.	5.00 (5.00; 5.00)		Paracetamol^d^; Dipyrone 500–1000 mg q6hr or q8hr^c,d^ h^c,d^; Non-pharmacological treatment (e.g., physiotherapy, acupuncture, thermotherapy, electrostimulation, therapeutic massage)^c,d^ Analgesics (dipyrone or paracetamol) in combination with weak opioids (Tramadol ou Codeine)^c,d^	x^h^		x^g^
**Tramadol**		4.78 (4.44; 5.12)	In patients older than 75 years, a daily dose of over 300 mg is not recommended. Start with 12.5 mg q8hr, with progressive increases of 12.5 mg every 8hr (non-sustained-release) Maximum dose: 100 mg q8hr. Reduce dose and extend the dosing interval for patients with severe renal failure (Creatinine Clearance < 30 mL/min)d	Paracetamol^d^; Dipyrone 500–1000 mg q6hr or q8hr^c,d^; Non-pharmacological treatment (e.g., physiotherapy, acupuncture, thermotherapy, electrostimulation, therapeutic massage)^c,d^			x

^a^Confidence interval; ^b^Non-steroidal anti-inflammatory drugs; ^c^Suggestions from experts; ^d^Items validated only in the second round; ^e^Prolonged use of NSAIDs nonselective COX-2 increases risk of GI bleeding and peptic ulcer disease in high-risk groups, including those aged >75 or taking oral or parenteral corticosteroids, anticoagulants, or antiplatelet agents. ^f^Regular opiate use for more than 2 weeks in those with chronic constipation without concurrent use of laxatives; ^g^Risk of falls, fractures, confusion, dependency and withdrawal syndrome; ^h^Not effective oral analgesic in dosages commonly used; may have higher risk of neurotoxicity, including delirium, than other opioids; safer alternatives available; ^i^All dose adjustment/special considerations of use and the alternative therapies described in this table were validated by expert consensus (lower limit of confidence interval ≥ 4.0); ^j^Screening Tool to Alert doctors to Right Treatment; lEuropean Union.

A consensus also was reached for eight medication/medication classes that should be avoided in older patients with 16 different conditions or diseases (**Table 2**). The use of NSAIDs was considered inappropriate for seven different conditions (long term use in osteoarthritis and gout, history of peptic ulcer disease or gastrointestinal bleeding, hypertension, heart disease, and chronic kidney disease stage IV and V or estimated glomerular filtration rate <50 ml/min per 1.73 m^2^) while the use of corticosteroids was considered inappropriate in five different conditions (rheumatoid arthritis, osteoarthritis, osteoporosis, diabetes, and delirium).

**Table 2:** Potentially inappropriate medication use in the older patients considering diagnoses or conditions.

Inappropriate Medication	Disease/Condition	Concern	Average of Likert scales (CI95%)^a^ from panel members	Alternative drugs and/or therapies^e^	Concern described in other PIM lists		
					Beers	STOPP^d^	Eu^e^ (7) - PIM list
**NSAIDs** **^b^**	**Osteoarthritis**	Avoid the long-term use of NSAIDs^b^ (> 3 months) for symptom relief of osteoarthritis pain where safe alternatives are available.	5.00 (5.00; 5.00)^d^	Paracetamol; Dipyrone 500–1000 mg q6hr or q8hr^,d^; Non-pharmacological treatment (e.g., physiotherapy, acupuncture, thermotherapy, electrostimulation, and therapeutic massage)^c,d^	x^j^	x	
	**Gout**	Avoid the long-term use of NSAIDs^b^ (> 3 months) for chronic treatment of gout where there is no contraindication to a xanthine-oxidase inhibitor e.g. allopurinol.	4.78 (4.44; 5.12)	Paracetamol; Dipyrone 500–1000 mg q6hr or q8hr,^c,d^; Non-pharmacological treatment (e.g., physiotherapy, acupuncture, thermotherapy, electrostimulation, and therapeutic massage)^c,d^	x^j^	x	
	**History of peptic ulcer disease or gastrointestinal bleeding**	History of peptic ulcer disease or gastrointestinal bleeding (unless with concurrent PPI): Risk of peptic ulcer and gastrointestinal bleeding relapse.	4.89 (4.63; 5.15)	Paracetamol; Dipyrone 500–1000 mg q6hr or q8hr,^c,d^; Non-pharmacological treatment (e.g., physiotherapy, acupuncture, thermotherapy, electrostimulation, and therapeutic massage)^c,d^	x^f^	x^f^	
	**Hypertension**	Risk of exacerbation of hypertension.	4.67 (4.12; 5.21)	Paracetamol; Dipyrone 500–1000 mg q6hr or q8hr^c,d^; Non-pharmacological treatment (e.g., physiotherapy, acupuncture, thermotherapy, electrostimulation, and therapeutic massage)^c,d^		x	
**NSAIDs** **^b^**	**Heart failure**	Potential to promote fluid retention and exacerbate heart failure.	4.78 (4.44; 5.12)	Paracetamol; Dipyrone 500–1000 mg q6hr or q8hr,^c,d^; Non-pharmacological treatment (e.g., physiotherapy, acupuncture, thermotherapy, electrostimulation, and therapeutic massage)^c,d^	x	x	
	**Chronic kidney disease Stages IV or less (creatinine clearance <30 ml/min)**	May increase risk of acute kidney injury and further decline of renal function.	5.00 (5.00; 5.00)	Paracetamol; Dipyrone 500–1000 mg q6hr or q8hr,^d^; Non-pharmacological treatment (e.g., physiotherapy, acupuncture, thermotherapy, electrostimulation, and therapeutic massage)^c,d^	x		
	**eGFR < 50 ml/min/1.73m** **^2^**	NSAIDs^b^ if eGFR < 50 ml/min/1.73m^2^: risk of deterioration in renal function.	5.00 (5.00; 5.00)	Paracetamol; Dipyrone 500–1000 mg q6hr or q8hr,^d^; Non-pharmacological treatment (e.g., physiotherapy, acupuncture, thermotherapy, electrostimulation, and therapeutic massage)^c,d^		x	
**COX-2-selective NSAIDs** **^b^**	**Cardiovascular disease**	COX-2 selective NSAIDs with concurrent cardiovascular disease (increased risk of myocardial infarction and stroke).		Paracetamol^d^; Dipyrone 500–1000 mg for q6hr or q8hr,^c,d^; Non-pharmacological treatment (e.g., physiotherapy, acupuncture, thermotherapy, electrostimulation, and therapeutic massage)^c,d^		x	
	Celecoxib		4.83 (4.40; 5.26)^d^			x	
	Etoricoxib		4.67 (4.28; 5.05)				
**Orphenadrine and Cyclobenzaprine**							
	**Delirium**	Avoid in older adults with or at high risk of delirium because of the potential of inducing or worsening delirium.	5.00 (5.00; 5.00)	Non-pharmacological treatment (e.g., physiotherapy, acupuncture, thermotherapy, electrostimulation, and therapeutic massage)^c,d^	x^g^		
	**Dementia or cognitive impairment**	Avoid because of adverse CNS Effects.	5.00 (5.00; 5.00)	Non-pharmacological treatment (e.g., physiotherapy, acupuncture, thermotherapy, electrostimulation, and therapeutic massage)^c,d^	x^g^		
	**Lower urinary tract symptoms. benign prostatic hyperplasia:**	May decrease urinary flow and cause urinary retention. Avoid in men.	4.78 (4.44; 5.12)	Non-pharmacological treatment (e.g., physiotherapy, acupuncture, thermotherapy, electrostimulation, and therapeutic massage)^c,d^	x^g^		
**Colchicine**							
	**Gout**	Avoid the long-term use of colchicine for chornic treatment of gout where there is no contraindication to a xanthine-oxidase inhibitor e.g. allopurinol.	4.83 (4.40;5.26)^d^	Paracetamol^c,d^; Dipyrone 500–1000 mg q6hr or q8hr,^c,d^; Non-pharmacological treatment (e.g., physiotherapy, acupuncture, thermotherapy, electrostimulation, and therapeutic massage)^c,d^		x	
	**eGFR < 10 ml/min/1.73m2 or creatinine clearance <30 ml/min)**	Risk of colchicine toxicity; Higher risk of gastrointestinal, neuromuscular, bone marrow adverse effects Toxicity.	4.78 (4.44; 5.12)	Paracetamol^c,d^; Dipyrone 500–1000 mg q6hr or q8hr,^c,d^; Non-pharmacological treatment (e.g., physiotherapy, acupuncture, thermotherapy, electrostimulation, and therapeutic massage)^c,d^	x^h^	x	
**Corticosteroids**							
	**Rheumatoid arthrtitis**	Long-term corticosteroids (> 3 months) as monotherapy for rheumatoid arthritis: Safer alternatives available; unnecessary exposure to systemic corticosteroid side-effects.	4.89 (4.63; 5.15)			x	
	**Osteoarthritis**	Safer alternatives available; unnecessary exposure to systemic corticosteroid side-effects.	4.78 (4.27; 5.29)			x	
	**Osteoporosis** **^c^**	long - term use of corticosteroids may increase bone loss and worsen osteoporosis.	5.00 (5.00; 5.00)^d^				
	**Diabetes** **^c^**	long - term corticosteroids may cause difficulty in controlling blood glucose level.	4.83 (4.40; 5.26)^d^				
	**Delirium**	Avoid in older adults with or at high risk of delirium because of the potential of worsening or inducing delirium.	4.78 (4.27; 5.29)		x		
**Opioids**							
	**History of falls or fractures**	May cause ataxia. impaired psychomotor function. syncope. additional falls.	4.56 (4.15; 4.96)	Paracetamol^c,d^; Dipyrone 500–1000 mg q6hr or q8hr,^c,d^; Non-pharmacological treatment (e.g., physiotherapy, acupuncture, thermotherapy, electrostimulation, and therapeutic massage)^c,d^	x	x	
**Pethidine/Meperidine**							
	**Delirium**	Avoid in older adults with or at high risk of delirium because of the potential of inducing or worsening delirium.	4.78 (4.27; 5.29)	Paracetamol^c,d^; Dipyrone 500–1000 mg q6hr or q8hr,^c,d^; Non-pharmacological treatment (e.g., physiotherapy, acupuncture, thermotherapy, electrostimulation, and therapeutic massage)^c,d^. Analgesics (dipyrone or paracetamol) in combination with weak opioids (tramadol or codeine)^c,d^.	x	x	
**Tramadol**							
	**Chronic seizures or epilepsy**	Lowers seizure threshold.	4.63 (4.19; 5.16)	Paracetamol^c,d^; Dipyrone 500–1000 mg q6hr or q8hr,^c,d^; Non-pharmacological treatment (e.g., physiotherapy, acupuncture, thermotherapy, electrostimulation, and therapeutic massage)^c,d^	x	x	

^a^Confidence interval; ^b^Non-steroidal anti-inflammatory drugs; ^c^Suggestions from experts; ^d^Items validated only in the second round; ^e^The alternative therapies described in this table were validated by expert consensus (lower limit of confidence interval ≥ 4.0); ^d^Screening Tool to Alert doctors to Right Treatment; ^e^European Union; ^f^ Non-COX-2 selective non-steroidal anti-inflammatory drug (NSAID) with history of peptic ulcer disease or gastrointestinal bleeding, unless with concurrent PPI or H2 antagonist (risk of peptic ulcer relapse; ^g^Medications classified as anticholinergic drugs in the criteria; ^h^ Used the measure Creatinine Clearance <30 ml/min; ^i^Long-term colchicine for treatment of gout where there is no contraindication to allopurinol. Allopurinol is first choice prophylactic drugs in gout; ^j^Avoid the long-term use of Non-COX-2 selective non-steroidal anti-inflammatory (> 3 months).

We presented the concerns also described in other PIM lists such as Beers (American Geriatrics Society Beers Criteria Update Expert P., 2015), STOPP criteria (O’Mahony et al., 2015) and the EU(7) PIM list (Renom-Guiteras et al., 2015) (**Tables 1** and **2**). Among all concerns validated in this study, about 65% were also reported in Beers consensus (American Geriatrics Society Beers Criteria Update Expert P, 2015). Lower consistency was observed when our list was compared with the STOPP criteria (47.2%) (O’Mahony et al., 2015) and the EU(7) PIM list (40.0%) (Renom-Guiteras et al., 2015).

In this study, the experts did not achieve consensus on 2/35 (5.7%) medication concerns regardless of diagnoses (phenylbutazone and tizanidine), on the use of strong opioids as first-line therapy for mild pain, and 12/23 (52.2%) dose adjustment/special considerations for medication use (i.e. special considerations for meperidine, indomethacin, and baclofen use) (**Appendix I**). The panel also did not reach a consensus about 35/63 (55.5%) alternatives therapies. These included medications such as ibuprofen, naproxen, and weak opioids (**Appendix II**).

## Discussion

The consensus method allowed us to identify PIM criteria and alternative treatment options for the treatment of pain and inflammation in older people, adapted to the Brazilian context. The panel reached consensus on 94 items which contain important information about the use of medicines for the treatment of pain and inflammation in older adults. Although these criteria were not designed to replace clinical judgement, PIM, and alternative therapy lists can be useful in informing prescribers, pharmacists, and health care planners (Hanlon et al., 2015), and may serve as a starting point for the safe and effective use of medications to treat pain and inflammation in older people. To the best of our knowledge, this is the first study in which a consensus on alternative therapies to PIMs adapted to the Brazilian context was reached.

In the absence of a strong evidence to guide the optimization of medication regimens in elderly people, the consensus of experts has been used as a strategy to develop PIM lists in several countries. The elaboration of PIM lists consists of a complex, dynamic, and time-consuming process which involves the combination of systematic reviews and expert opinion. For these reasons, many researchers have combined two or more tools and added other medications that they considered were missing in order to develop and adapt existing criteria to different settings (Kim et al., 2010; Chang et al., 2012; Renom-Guiteras et al., 2015; Kim et al., 2015; Oliveira et al., 2016). Our list was based on the Beers criteria (American Geriatrics Society Beers Criteria Update Expert P, 2015), STOPP criteria (O’Mahony et al., 2015), and the EU(7) PIM lists published in 2015 (Renom-Guiteras et al., 2015). Although this last list has not been widely used compared with the Beers and STOPP criteria, it included some drugs rarely included in other PIM lists and therapeutic alternatives.

We have compared the results obtained in this consensus with previous lists: Beers criteria (American Geriatrics Society Beers Criteria Update Expert P, 2015), STOPP criteria (O’Mahony et al., 2015), EU(7) PIM list (Renom-Guiteras et al., 2015), and Brazilian PIM list (Oliveira et al., 2016). Although the list developed by Oliveira et al. (Oliveira et al., 2016) was based on previous versions of Beers (2012) (American Geriatrics Society Beers Criteria Update Expert P, 2012) and STOPP (2008) (Gallagher et al, 2008), it was the first Brazilian PIM list. Thus, the comparison between our list and previous explicit criteria may contribute to improving the knowledge on PIMs and alternative therapies which had not been previously investigated in the Brazilian setting.

In our study, consensus in the Delphi process was reached for 95% of the concerns related to the use of medications for the treatment of pain and inflammation in elderly individuals. In contrast with Beers criteria (American Geriatrics Society Beers Criteria Update Expert P., 2015) and the Brazilian PIM list (Oliveira et al., 2016), all NSAIDs were considered to be PIMs, regardless of the diagnosis, in our study. These results may be justified by the fact that our experts suggested and validated alternative therapies for these medications. They considered that one approach to reducing adverse drug reactions associated with NSAIDs is to avoid the use of these medications and use preferred alternative therapies, especially in those older adults with pre-existing diseases. There are few data informing the use of these medications in elderly who frequently have additional comorbidities and use multiple medications which increase the risk of adverse effects (Reid et al., 2011; Makris et al., 2017). On the other hand, for those patients that require an NSAID even after the use of alternative therapies, the experts also reached consensus that some NSAID should be used at the lowest effective dose. In addition, this panel incorporated two new concerns about the use of corticoids in older people who have diagnosed with osteoporosis and diabetes. These concerns were also presented in other previously published PIM lists (Winit-Watjana et al., 2008; Kim et al., 2010).

Some concerns about medications only achieved consensus in the second round of questioning. In these cases, there was doubt about the evidence concerning the increased risk of adverse effects in elderly patients. For example, we can cite the concern about the use of celecoxib in older patients diagnosed with cardiovascular diseases, where there is conflicting data from different randomised controlled trials (Solomon et al., 2005; Nissen, 2017).

A consensus could not be reached on two concerns (phenylbutazone and tizanidine) regardless of diagnosis even after the second round. These medications were also not described as PIMs independent of diagnosis in the Brazilian list published during the Delphi process (Oliveira et al., 2016). This result may be explained by the lack of experience regarding the use of these medications in the Brazilian context, since they are not included in the national list of essential medications (RENAME) available at no cost and are thus seldom prescribed in this setting (Ministry of Health, Secretariat of ScienceTechnology and Strategic Inputs. [National List of Essential Medicines: RENAME 2017]: Ministry of Health, 2017). Regarding PIM in specific disease or conditions, the concern about the use of strong oral or transdermal opioids as first-line therapy for mild pain, also did not achieve consensus; it received the lowest media in this study. The appropriate pain assessment in older patients may be complex. Barriers such as underreporting of pain by patients and the presence of cognitive deficits and comorbidities may complicate this evaluation in older people (Horgas, 2017; Schofield, 2018). Thus, some experts may have considered that the prescription of strong opioids should incorporate individualized clinical judgement, and that a generalised statement would not be appropriate.

In contrast to other lists, the recommendation about the combined use of NSAIDs and proton pump inhibitors (PPIs) did not reach a consensus in this study. This result is also because the experts did not think NSAIDs should be used in older people. Furthermore, during the consensus process, the experts were concerned that the prescription of drugs such as PPIs represents increasing drug burden which may also put individuals at risk of other adverse events such as fractures and *Clostridium difficile* infection. Studies showed that *the* co-administration of PPIs does not prevent NSAID-induced intestinal damage but might actually aggravate it (Scarpignato et al., 2016). Thus, this result suggests that specialists prefer to use alternative therapies rather than use PPIs to avoid problems related to the use of NSAIDs for the treatment of pain and inflammation.

The panel has also addressed possible alternative therapies which may be prescribed to treat pain and inflammation. Paracetamol (acetaminophen) and dipyrone (metamizole) were identified as possible alternative therapies by experts in several concerns. Although recent literature has demonstrated the limited efficacy of paracetamol (Marcum et al., 2016), it remains recommended in guidelines as a first-line pharmacologic treatment for older adults with mild-to-moderate pain (American Geriatrics Society Panel on Pharmacological Management of Persistent Pain in Older P, 2009; Abdulla et al., 2013). With its dose-dependent hepatotoxicity, the recommended maximum daily doses should not be exceeded (3250 mg) and it should be prescribed with caution (lower doses) in patients with pre-existing liver disease, malnutrition, anorexia, heavy alcohol intake, or in patients treated by hepatic enzyme inducers (rifampicin, phenytoin, carbamazepine, or barbiturates) (Yoon et al., 2016). Regarding the use of dypirone, studies have demonstrated that for short term use, this medication appeared to be a safe choice when compared to other analgesics. However, the intermediate and long term safety of dypirone are still not well documented (Kotter et al., 2015; Andrade et al., 2016).

Interestingly, the experts agree strongly about the use of non-pharmacological interventions such as physiotherapy, acupuncture, thermotherapy, electrostimulation, and therapeutic massage as alternative therapies for the treatment of pain and inflammation in older people. Nonpharmacological approaches can help avoid drugs that have high risks of causing adverse events. For this reason, the body of evidence about nonpharmacological approaches is growing in older adults, especially, in persons with dementia and delirium (Livingston et al., 2014; Resnick et al., 2014; Hshieh et al., 2015).

The internet-based Delphi method offers a practical and cost-effective approach to identifying areas of concordance and disagreement involving a geographically dispersed group of experts. Another advantage of this method is the anonymity that encourages experts to make statements on the basis of their personal knowledge and experience. Finally, a significant advantage of the Delphi method is that participants are very much aware at each stage of the results of the previous rounds, and there is scope for each expert to provide more detailed feedback on both the process and the results. In this study, the majority of alternative therapies were suggested by the experts. Some of these differ from other previously published PIM lists (Renom-Guiteras et al., 2015; American Geriatrics Society Beers Criteria Update Expert P, 2015; O’Mahony et al., 2015). This result demonstrated the importance of set-specific country lists. In this study, we selected the most comprehensive and updated lists published based on a literature review (Motter et al., 2018). To the best of our knowledge, to date, this study is the first to validate alternative therapies to PIMs in a Brazilian setting.

In order to minimise the inclusion of controversial PIMs or alternative therapies, we decided before the development of the consensus process that an item should be included in the final list only if the lower limit of the 95% CI was ≥ 4.0. Thus, not only the mean score was taken into consideration but also the degree of discord.

There are, however, some limitations which must be acknowledged in this study. Thus, the results must be interpreted with caution. Firstly, although we carefully searched for experts who comprised the consensus process panel, the limited participation of experts and the drop out of two participants in the second round may have compromised the representativeness of some areas of expertise and geographic regions. Secondly, the lack of engagement of experts in the second round may restrict the application of the Delphi method. Finally, the authors did not perform an evaluation of the quality and strength of evidence for each concern or alternative therapies presented in this study. The items were based on information available in some consensus publications referred to. These tools were published in 2015 and new findings from recent clinical trials or systematic review (Beers PIM list was updated in 2019) were not reviewed; thus, we recognize that some PIMs might have been added to or excluded from the next version.

## Conclusions

Explicit criteria summarized specific statements for identifying problems with medications in older people which make drugs easy to use by clinicians and health professionals. Condition-specific country lists are an important tool which may improve the rational use of medications among older people. In this study, we reached a consensus on 94 items that contain important information about the use of medicines used in the treatment of pain and inflammation in older adults. We believe that the application of our criteria combined with clinical judgement should contribute to helping physicians, pharmacists, and other health professionals to optimize the treatment of pain and inflammation in older patients. In this study, we also included special considerations of use and therapeutic alternatives; these may be an important addition to the screening process in caring for elderly Brazilian people. Future research should evaluate the implementation of the list among health professionals, including the usefulness of the suggestions for special considerations of medication use and alternative therapies, and apply this methodology to other therapeutic areas.

## Data Availability Statement

All datasets generated for this study are included in the article/**Supplementary Material**.

## Ethics Statement

The studies involving human participants were reviewed and approved by Ethics Committee of Universidade do Vale do Rio dos Sinos (UNISINOS) (number: 1.731.392). The patients/participants provided their written informed consent to participate in this study.

## Author Contributions

FM and VP participated in all stages of this research, from the design and interpretation of data to the final write-up. FM prepared the work documents during the development process, recruited the experts and coordinated the delphi survey. FM, VP, and SH assisted with interpretation of the data and preparation of the final version of PIM list. FM drafted the manuscript, supported by VP and SH. All the authors critically reviewed and approved the final manuscript.

## Funding

FM was supported by the coordination for the improvement of Higher Education Personnel – CAPES through a doctorate at University of Vale do Rio dos Sinos, Brazil (grant number: 88887159088/2017-00). FM was also supported by CAPES through a sandwich doctorate fellowship at The University of Sydney, Australia (grant number: 88881.134589/2016-01). This systematic review was funded by the National Council for Scientific and Technological Development-CNPQ (grant number: 426720/2016-4). The funders were not involved in the design or conduct of the study, collection, analysis, or interpretation of the data or preparation or approval of the manuscript.

## Conflict of Interest

The authors declare that the research was conducted in the absence of any commercial or financial relationships that could be construed as a potential conflict of interest.
