# Supplementary material for: Pain and Inflammation Management in Older Adults: A Brazilian Consensus of Potentially Inappropriate Medication and Their Alternative Therapies
Source: Front Pharmacol. 2019 Dec 2;10:1408. doi: 10.3389/fphar.2019.01408 (PMC6901010; doi:10.3389/fphar.2019.01408)
Supplement: Supplementary file 1 [file DataSheet_1.docx]

| **Appendix 1. Inappropriate medication for older patients independent of diagnosis, inappropriate medication for older patients dependent on diagnosis, and dose adjustment/ special considerations of use that did not reach consensus after the second round.** | | | | | | | |
| --- | --- | --- | --- | --- | --- | --- | --- |
| **Medication/Medication class** | **Description** | **Average of Likert**  **scales (CI 95%)^a^ from panel members** | **Item described in other PIM lists** | | | | |
|  |  |  | **Beers 2015** | **STOPP^b^ 2015** | **Eu^c^(7) - PIM list** | |  |
| **Inappropriate medication for older patients independent of diagnosis** | | |  |  | |  |  |
| Phenylbutazone | Risk of blood dyscrasia | 4.57 (3.84; 5.30) |  |  | | x |  |
| Tizanidine | Most muscle relaxants poorly tolerated by older adults because some have anticholinergic adverse effects; sedation; increased risk of fractures; effectiveness at dosages tolerated by older adults questionable. | 4.57 (3.84; 5.30) |  |  | | x |  |
| **Inappropriate medication for older patients dependent on diagnosis** | |  |  |  | |  |  |
| Opioids | Use of oral or transdermal strong opioids as first-line therapy for mild pain (WHO^f^ analgesic ladder not observed). | 2.43 (1.03; 3.83) |  | x | |  |  |
| **Dose adjustment/special considerations of use** | |  |  |  | |  |  |
| NSAIDs^d^ | The risk of bleeding may be reduced if combined with proton-pump inhibitors (use <8 weeks) | 4.23 (3.59; 4.98) | x | x | | x |  |
| Ketoprofen | Dose should be reduced if creatinine clearance <20 ml/min. | 4.43 (3.03-5.83) |  |  | | x |  |
| Indomethacin | Reduce dose by 25% | 4.14 (3.25; 5.13) |  |  | | x |  |
| Muscle relaxants | Assessment of renal function prior to prescription, especially for long-term use^e^. | 4.67 (3.81; 5.52) |  |  | |  |  |

| **Appendix 1. Inappropriate medication for older patients independent of diagnosis, inappropriate medication for older patients dependent on diagnosis, and dose adjustment/ special considerations of use that did not reach consensus after the second round (continued).** | | | | | | |
| --- | --- | --- | --- | --- | --- | --- |
| **Medication/Medication class** | | **Description** | **Average of Likert**  **scales (CI 95%)^a^ from panel members** | **Item described in other PIM lists** | | |
|  |  |  |  | **Beers 2015** | **STOPP^b^ 2015** | **Eu^c^(7) - PIM list** |
| **Dose adjustment/special considerations of use (continued)** | | | |  |  |  |
| Baclofen | | Start with 5 mg 2–3 times daily and increase gradually as needed. | 4.00 (3.84; 5.30) |  |  | x |
|  |  | Maximum dose: 10 mg 3 times daily. | 4.00 (3.84; 5.30) |  |  | x |
| Pethidine/Meperidine | | Use for the shortest period possible. | 4.14 (2.69; 5.60) |  |  | x |
|  | | 50 mg every 4–6 hours | 3.71 (2.33; 5.10) |  |  | x |
|  | | Use 75% of the normal dose at the usual intervals in cases of moderate renal failure (GFR^g^ 10–50 ml/min) | 3.86 (2.50; 5.21) |  |  | x |
|  | | Use 50% of the normal dose at the usual intervals in cases of severe renal failure (GFR^g^ <10 ml/min) | 3.86 (2.50; 5.21) |  |  | x |
| Tramadol | | Use tramadol (reduced dose) in combination with dipyrone^e^. | 4.42 (3.70; 5.16) |  |  |  |
|  | | Preferential presentation in drops. This will allow to define the most effective dose in terms of pain and with a lower number or intensity of side effects^.e^ | 4.14 (3.31; 4.97) |  |  |  |

^a^Confidence interval; ^b^Screening Tool to Alert doctors to Right Treatment; ^c^European Union; ^d^Non-steroidal anti-inflammatory drugs; ^e^Suggestions from experts; ^f^World Health Organization; ^g^Glomerular Filtration Rate
