# Supplementary material for: Pain and Inflammation Management in Older Adults: A Brazilian Consensus of Potentially Inappropriate Medication and Their Alternative Therapies
Source: Front Pharmacol. 2019 Dec 2;10:1408. doi: 10.3389/fphar.2019.01408 (PMC6901010; doi:10.3389/fphar.2019.01408)
Supplement: Supplementary file 2 [file DataSheet_2.docx]

| **Appendix 2. Alternative therapies did not reach consensus after the second round** | | |  |
| --- | --- | --- | --- |
| **Medication/Medication class** | **Alternative therapies** | **Average of Likert**  **scales (CI95%)^a^ from panel members** | **Alternative therapies described in Eu^e^(7) - List** |
| **NSAIDs^b^** | Ibuprofen (≤3 x 400 mg/d or for a period shorter than one week) | 3.86 (2.87; 4.86) | x |
|  | Naproxen (≤2 x 250 mg/d or for a period shorter than one week) | 4.29 (3.59; 4.98) | x |
|  | Opioids with lower risk of delirium | 4.29 (3.59; 4.98) | x |
|  | Topical therapies (NSAIDs^b^. capsaicin. menthol. lidocaine)^d^ | 4.29 (3.59; 4.98) |  |
| **COX-2^c^selective NSAIDs^b^** | Ibuprofen (≤3 x 400 mg/d or for a period shorter than one week) | 3.86 (2.50; 5.21) | x |
|  | Naproxen (≤2 x 250 mg/d or for a period shorter than one week) | 3.71 (2.83; 4.59) | x |
|  | Opioids with lower risk of delirium | 4.14 (3.79; 4.49) | x |
|  | Topical therapies (NSAIDs^b^. capsaicin. menthol. lidocaine)^d^ | 4.29 (3.59; 4.98) |  |
| **Phenylbutazone** | Paracetamol | 4.43 (3.38; 5.48) | x |
|  | Ibuprofen (≤3 x 400 mg/d or for a period shorter than one week) | 3.86 (2.87; 4.85) | x |
|  | Naproxen (≤2 x 250 mg/d or for a period shorter than one week) | 4.17 (3.38; 4.96) | x |
|  | Opioids with lower risk of delirium | 4.14 (3.79; 4.49) | x |
|  | Topical therapies (NSAIDs^b^. capsaicin. menthol. lidocaine)^d^ | 4.29 (3.59; 4.98) |  |
| **Indomethacin** | Ibuprofen (≤3 x 400 mg/d or for a period shorter than one week) | 3.86 (2.87; 4.86) | x |
|  | Naproxen (≤2 x 250 mg/d or for a period shorter than one week) | 3.71 (2.69; 4.74) | x |
|  | Opioids with lower risk of delirium | 4.14 (3.79; 4.49) | x |
|  | Topical therapies (NSAIDs^b^. capsaicin. menthol. lidocaine)^d^ | 4.29 (3.59; 4.98) |  |
| Ketorolac | Ibuprofen (≤3 x 400 mg/d or for a period shorter than one week) | 3.71 (2.69; 4.74) | x |
|  | Naproxen (≤2 x 250 mg/d or for a period shorter than one week) | 3.71 (2.69; 4.74) | x |
|  | Opioids with lower risk of delirium | 4.00 (3.47; 4.53) | x |
|  | Topical therapies (NSAIDs^b^. capsaicin. menthol. lidocaine)^d^ | 4.29 (3.59; 4.98) |  |
|  |  |  |  |

| **Appendix 2. Alternative therapies did not reach consensus after the second round (continued)** | | |  |
| --- | --- | --- | --- |
| **Medication/Medication class** | **Alternative therapies** | **Average of Likert**  **scales (CI95%)^a^ from panel members** | **Alternative therapies described in Eu^e^(7) - List** |
| **Muscle Relaxants** | Analgesics (dipyrone or paracetamol) in combination with weak opioids (Tramadol ou Codeine)^d^ | 4.24 (3.15;5.13) |  |
|  | Dipyrone 500 - 1000mg for 6/6 hours or 8/8 hours^d^ | 4.14 (3.15-5.13) |  |
|  | Botulinum toxin in combination with Physiotherapy in frail elderly^d^ | 4.43 (3.53;5.33) | x |
| **Colchicine** | Ibuprofen (≤3 x 400 mg/d or for a period shorter than one week) | 3.86 (2.87; 4.86) | x |
|  | Naproxen (≤2 x 250 mg/d or for a period shorter than one week) | 3.86 (2.87; 4.86) | x |
|  | Oral corticosteroids ^d^ |  |  |
|  | Weak opioids^d^ | 4.43 (3.93; 4.92) |  |
|  | Analgesics (dipyrone or paracetamol) in combination with weak opioids (Tramadol or Codeine)^d^ | 4.29 (3.59; 4.98) |  |
| **Pethidine/Meperidine** | Ibuprofen (≤3 x 400 mg/d or for a period shorter than one week) | 3.57 (2.39; 4.78) | x |
|  | Naproxen (≤2 x 250 mg/d or for a period shorter than one week) | 3.86 (2.87; 4.84) | x |
|  | Opioids with lower risk of delirium | 4.43 (3.93; 4.92) | x |
| **Tramadol** | Ibuprofen (≤3 x 400 mg/d or for a period shorter than one week) | 3.86 (2.87; 4.86) | x |
|  | Naproxen (≤2 x 250 mg/d or for a period shorter than one week) | 3.86 (2.87; 4.86) | x |
|  | Opioids with lower risk of delirium | 4.43 (3.93; 4.92) | x |

^a^Interval of confidence; ^b^Non-steriodal anti-inflammatory drugs; ^c^ Cyclooxygenase-2; ^d^Sugestions from experts; ^e^European Union.
