# Supplementary material for: Pain and Inflammation Management in Older Adults: A Brazilian Consensus of Potentially Inappropriate Medication and Their Alternative Therapies
Source: Front Pharmacol. 2019 Dec 2;10:1408. doi: 10.3389/fphar.2019.01408 (PMC6901010; doi:10.3389/fphar.2019.01408)
Supplement: Supplementary file 3 [file DataSheet_3.docx]

| **APPENDIX 3. COMPARATION OF PIM LIST POTENTIALLY INAPPROPRIATE DRUGS FOR THE OLDER PATIENTS VALIDATED BY EXPERT CONSENSUS WITH BRAZILIAN PIM LIST [38]**  **Table1. Potentially inappropriate drugs for the older patients independent of diagnosis validated by expert consensus** | | | | | | |
| --- | --- | --- | --- | --- | --- | --- |
| **Inappropriate medication** | | **Concern** | **Concern described in**  **Brazilian PIM list [38]** | | | |
| **NSAIDs**^b^ | Diclofenac | Very high risk of gastrointestinal bleeding, ulceration, or perforation, which may be fatal. | x^e^ | | | |
|  | Etodolac |  |  |  |  |  |
|  | Aceclofenac |  |  |  |  |  |
|  | Piroxicam |  |  |  |  |  |
|  | Lornoxicam |  |  |  |  |  |
|  | Tenoxicam |  |  |  |  |  |
|  | Meloxicam |  |  |  |  |  |
|  | Ibuprofen |  |  |  |  |  |
|  | Flurbiprofen |  |  |  |  |  |
|  | Loxoprofen |  |  |  |  |  |
|  | Mefenamic acid |  |  |  |  |  |
|  | Celecoxib |  |  |  |  |  |
|  | Etoricoxib |  |  |  |  |  |
|  | Nimesulide |  |  |  |  |  |
|  | Acetylsalicylic acid |  |  |  |  |  |
|  | Phenylbutazone |  |  |  |  |  |
|  | Indomethacin |  |  |  |  |  |
|  | Ketorolac |  |  |  |  |  |
|  | Naproxen |  |  |  |  |  |
|  | Ketoprofen |  |  |  |  |  |
| **Indomethacin** | | Indomethacin is more likely than other NSAIDs^b^ to have adverse central nervous system effects. |  |  | **x** |  |
| **Ketorolac** | | Increased risk of acute kidney injury in older adults. |  |  |  |  |
| **Ibuprofen** | | Ibuprofen (>3 × 400 mg/day): increased risk of cardiovascular complications at higher doses (>1200 mg/day), especially in cases of previous cardiovascular disease. |  |  |  |  |
| **Acetylsalicylic acid** | | Acetylsalicylic acid (>325 mg): increased risk of bleeding due to prolonged clotting time, elevation of INR values or inhibition of platelet aggregation. |  |  |  |  |
| **Tramadol** | | More adverse effects in older adults; CNS side effects such as confusion, vertigo, and nausea. |  |  |  |  |
|  | | | | | | |
| **Muscle relaxants**  Carisoprodol  Orphenadrine  Baclofen  Thiocolchicoside  Cyclobenzaprine | | Most muscle relaxants are poorly tolerated by older adults owing to their anticholinergic adverse effects, sedation, and increased risk of fractures; their effectiveness at dosages tolerated by older adults is questionable. |  |  | **x**  **x**  **x**  **x** |  |
| **Colchicine** | | Higher risk of toxicity in older adults, particularly in cases of existing renal, gastrointestinal infections, or cardiac disease. |  |  |  |  |
| **Opioids** | | Use of regular (as distinct from PRN) opioids without concomitant laxative confers a risk of severe constipation. |  |  | x^f^ |  |
| **Meperidine/ Pethidine** | | Risk of falls, fractures, confusion, dependency and withdrawal syndrome. Not effective oral analgesic in dosages commonly used. May have higher risk of neurotoxicity (including delirium) than other opioids. |  |  | x^h^ |  |

| **Table2. Potentially inappropriate medication use in the older patients considering diagnoses or conditions** | | | |
| --- | --- | --- | --- |
| **Inappropriate Medication** | **Disease/Condition** | **Concern** | **Concern described in**  **Brazilian PIM list [38]** |
| **NSAIDs^b^** | **Osteoarthritis** | Avoid the long-term use of NSAIDs^b^  (>3 months) for symptom relief of osteoarthritis pain where safe alternatives are available. | **x^j^** |
|  | **Gout** | Avoid the long-term use of NSAIDs^b^ (>3 months) for chronic treatment of gout where there is no contraindication to a xanthine-oxidase inhibitor e.g. allopurinol. | **x^j^** |
|  | **History of peptic ulcer disease or gastrointestinal bleeding** | History of peptic ulcer disease or gastrointestinal bleeding (unless with concurrent PPI): Risk of peptic ulcer and gastrointestinal bleeding relapse. | x |
|  | **Hypertension** | Risk of exacerbation of hypertension. | x |
|  | **Heart failure** | Potential to promote fluid retention and exacerbate heart failure. | x |
|  | **Chronic kidney disease Stages IV or less (creatinine clearance <30 mL/min)** | May increase risk of acute kidney injury and further decline of renal function. | x |
|  | **eGFR < 50 ml/min/1.73m^2^** | NSAIDs^b^ if eGFR < 50 ml/min/1.73m^2^: risk of deterioration in renal function. |  |
| **COX-2-selective NSAIDs^b^**  Celecoxib  Etoricoxib | **Cardiovascular disease** | COX-2 selective NSAIDs with concurrent cardiovascular disease (increased risk of myocardial infarction and stroke). | x  x |
| **Orphenadrine and Cyclobenzaprine** | **Delirium** | Avoid in older adults with or at high risk of delirium because of  the potential of inducing or worsening delirium. | x^g^ |
|  | **Dementia or cognitive impairment** | Avoid because of adverse CNS  Effects. | x |
|  | **Lower urinary tract symptoms. benign prostatic hyperplasia:** | May decrease urinary flow and cause urinary retention. Avoid in men. | x^g^ |
| **Colchicine** | **Gout** | Avoid the long-term use of colchicine for chornic treatment of gout where there is no contraindication to a xanthine-oxidase inhibitor e.g. allopurinol. | x^i^ |
|  | **eGFR < 10 ml/min/1.73m2 or creatinine clearance <30 mL/min)** | Risk of colchicine toxicity; Higher risk of gastrointestinal, neuromuscular, bone marrow adverse effects  Toxicity. |  |
| **Corticosteroids** | **Rheumatoid arthrtitis** | Long-term corticosteroids (>3 months) as monotherapy for rheumatoid arthritis: Safer alternatives available; unnecessary exposure to systemic corticosteroid side-effects. | x |
|  | **Osteoarthritis** | Safer alternatives available; unnecessary exposure to systemic corticosteroid side-effects. | x |
|  | **Osteoporosis^c^** | long - term use of corticosteroids may increase bone loss and worsen osteoporosis. |  |
|  | **Delirium** | Avoid in older adults with or at high risk of delirium because of the potential of worsening or inducing delirium. |  |
|  | **Diabetes^c^** | long - term corticosteroids may cause difficulty in controlling blood glucose level. | x |
| **Opioids** | **History of falls or fractures** | May cause ataxia. impaired psychomotor function. syncope. additional falls. | x |
| **Pethidine/Meperidine** | **Delirium** | Avoid in older adults with or at high risk of delirium because of the potential of inducing or worsening delirium. | x |
| **Tramadol** | **Chronic seizures or epilepsy** | Lowers seizure threshold. | x |
